# Preterm Delivery: Microbial Dysbiosis, Gut Inflammation and Hyperpermeability

**DOI:** 10.3389/fmicb.2021.806338

**Published:** 2022-02-04

**Authors:** Silvia Arboleya, David Rios-Covian, Flore Maillard, Philippe Langella, Miguel Gueimonde, Rebeca Martín

**Affiliations:** ^1^IPLA-CSIC, Paseo Río Linares, Villaviciosa, Spain; ^2^Paris-Saclay University, INRAE, AgroParisTech, Micalis Institute, Jouy-en-Josas, France

**Keywords:** neonatal period, gut permeability, gut inflammation, microbiota, preterm/full term infants

## Abstract

Preterm birth is one of the main health problems encountered in the neonatal period, especially because it is also the first cause of death in the critical 1st month of life and the second in children under 5 years of age. Not only preterm birth entails short term health risks due to low weight and underdeveloped organs, but also increases the risk of suffering from non-transmissible diseases in the long term. To date, it is known that medical conditions and lifestyle factors could increase the risk of preterm birth, but the molecular mechanisms that control this process remain unclear. Luteolysis, increased inflammation or oxidative stress have been described as possible triggers for preterm birth and, in some cases, the cause of dysbiosis in preterm neonates. Several murine models have been developed to shed light into the mechanistic of preterm birth but, for the most part, are inflammation-based labor induction models and the offspring health readouts are mainly limited to survival and weight. Using a set of SWISS-CD1 mice born prematurely we analyzed inflammation and gut permeability parameters compared with term pups at weaning age. Overall, preterm mice presented higher systemic inflammation and gastrointestinal tract permeability. In this perspective article, we discuss the recent discoveries on preterm birth and the necessity of non-inflammatory murine models to really understand these phenotypes and be able to design strategies to prevent the sequels of this traumatic event in neonates.

## Introduction: Preterm Birth Effect on Infant Health and Gut Dysbiosis

Preterm birth is defined by the World Health Organization as any birth before 37 completed weeks of gestation. It can be further divided based on gestational age (GA) as: extremely preterm (<28 weeks); very preterm (28 – < 32 weeks); moderate or late preterm (32 – < 37 completed weeks of gestation) (Howson and Lawn, 2012). Around 15 million babies are born preterm each year worldwide (∼10% of livebirths) and more than 500,000 births are premature in Europe, with different ranges among countries (from 5.5% in Ireland to 11.4% in Austria) (EPHR, 2008; Blencowe et al., 2012). In the light of these numbers, preterm infants represent the largest child patient group in Europe (Haumont et al., 2012). Approximately 1 million babies per year die due to complications linked to preterm birth. Preterm delivery is thus now considered the second leading cause of death in children under 5 years of age and the single most important cause of death in the critical 1st month of life (Haumont et al., 2012; Walani, 2020).

Early birth entails a huge range of short- and long-term health complications and disabilities in survivors. Preterm babies born with low weight, underdeveloped organs and they are more likely to have long-term neurodevelopmental and body function deficits, such as impairments on infant growth and weight gain, cerebral palsy, sensorial and motor disabilities, learning and behavioral disorders, cardiorespiratory problems, even an increased risk for accelerated brain aging was observed in young adults after premature birth. Moreover, they undergo an increased risk of falling in non-communicable diseases such as diabetes, obesity, asthma or mental disorders (Stoll et al., 2004; Sammallahti et al., 2017; Heinonen et al., 2018; Hedderich et al., 2021; Kosiecz et al., 2021).

Premature delivery can be considered as a syndrome with many causes and factors (Haumont et al., 2012; Ansari et al., 2021). Medical conditions (urinary tract or vaginal infections, chorioamnionitis, uterine or cervical abnormalities, high blood pressure or obesity), lifestyle factors (high stress levels and long working hours, no prenatal care or drug use) or demographic factors (ethnicity, delayed fertility, multiple birth rate by assisted fertility with advanced maternal age), among others, can affect the birth outcome (Haumont et al., 2012; Ansari et al., 2021). A recent study has shown that pathways related to the oxidative stress response and the inflammation are deregulated in the placenta of spontaneous preterm deliveries (Lien et al., 2021), indicating the presence of an altered fetal environment for these babies. Moreover, this oxidative stress has been linked to many diseases associated with prematurity (Sánchez-Illana et al., 2021) and inflammation has been linked to the pathogenesis of preterm delivery even prior to placenta development (Green and Arck, 2020). In addition, the presence of oxidative stress has also been proposed as putative factor involved in shaping the premature infant gut microbiota at early life (Arboleya et al., 2012b).

The interruption of the natural intrauterine development of immune and intestinal systems in the third trimester results in a systemic immaturity that strongly affects the correct establishment and development of the gut microbiota (Henderickx et al., 2019). Gestational age has been described as an important influencer of gut microbiota colonization (Aguilar-Lopez et al., 2021); moreover, a multifactorial set of environmental conditions further influence to aberrant gut microbiota development in premature babies. Hospital environment and medical procedures such as positive airway pressure generated by respiratory support, parenteral feeding, limited or difficult breast-feeding, antibiotic use, together with the higher rates of C-section deliveries in premature babies, imply the development of an intestinal microbiota quite different to term infants (Henderickx et al., 2019; Aguilar-Lopez et al., 2021). A delay in gut colonization and a decrease bacterial diversity has been repeatedly reported. In addition, higher levels of potential pathogenic microorganisms were present in the preterm gut microbiota, in comparison with full-term neonates (Rougé et al., 2010; Jacquot et al., 2011; Arboleya et al., 2012a). Different studies observed lower levels of anaerobic bacteria such as *Bifidobacterium* or *Bacteroides* genera, and predominance of facultative anaerobes like enterobacteria (*Escherichia* or *Klebsiella*), enterococci, some clostridia or lactobacilli at the 1st months of life in preterm babies (Wang et al., 2009; Rougé et al., 2010; Jacquot et al., 2011; Arboleya et al., 2012a, 2015; Barrett et al., 2013; Groer et al., 2014; Wandro et al., 2018). Distinct gut microbial profiles were observed in children born prematurely even up to 4 years of age (Fouhy et al., 2019), indicating long term effects. Moreover, lower short chain fatty acids concentrations were recurrently observed (Arboleya et al., 2012a, 2015). In this context, it has been hypothesized that intestinal microbiota dysbiosis makes premature babies particularly susceptible to develop complications with detrimental long-term effects on infant’s health (Neu, 2020; Cuna et al., 2021). It seems that during the 1st weeks of life there is an opportunity for microbiome modulation as preventive treatment for preterm birth. A recent study estimated that the cervicovaginal metabolome was mainly impacted by the variability individual-individual, but the microbiota composition is the second factor that affects it in preterm birth, being greater than other factors like GA, ethnicity, maternal age or body mass index (Pruski et al., 2021).

## Lessons From Murine Models: Inflammatory vs. Non-Inflammatory Preterm Birth

As previously described, preterm birth entails the exposure to several stresses during and after pregnancy that can affect the correct development of the neonate. In humans, spontaneous preterm births are difficult to predict and to prevent, which leaves the scientific community with limited options to unravel the multiple causes and mechanisms that lead to preterm labor (McCarthy et al., 2018). In this situation, and despite some physiological differences between mice and human, murine models have demonstrated a great potential, especially in the case of inflammation related preterm delivery (Green and Arck, 2020); (McCarthy et al., 2018).

Several models, consisting in different inflammatory molecules or generation of knockout mouse in key genes implicated in the inflammatory immune response, are already developed to induce preterm labor. They have been used to validate some compounds potentially preventing preterm delivery, such as naloxone, progesterone or corticoids (Romero et al., 2006; Arenas-Hernandez et al., 2019; Green and Arck, 2020; Galaz et al., 2021; Ginsberg et al., 2021). Lipopolysaccharide (LPS) stimulated infection is one of the most popular models to study inflammatory preterm birth, probably because of its low-cost and effectivity (Huang et al., 2021). Within this model, several inflammatory pathways and features have been profoundly studied during preterm labor, such as the implication of Interleukin-27 (IL-27) in the activation of extracellular signal-regulated kinases (ERK) pathway, which ultimately origins preterm labor (Huang et al., 2021). In this model, interventions with probiotics, such as *Lentilactobacillus kefiri* (Lk48), can impair the inflammatory process and diminish the rate of neonatal death (Ventimiglia et al., 2021). Other kind of interventions, like the activation of the gen Rev-erbα, implicated in circadian rhythms, can revert LPS induced preterm labor models (Cui et al., 2021).

Another group of molecules implicated in preterm labor are alarmins, proteins which usually trigger the TLR4-patwhay in a similar way that LPS does. Some studies have been able to provoke preterm labor by the administration of high mobility group box-1 (HGMB1) alarmin, which was also accompanied by higher neonatal death (Radnaa et al., 2021). The same model was used to propose the corticosteroid betamethasone as a potential preventive treatment for preterm labor (Galaz et al., 2021). IL-33 is produced by B cells and it is another alarmin that can cause preterm birth (Valeff et al., 2020). In addition, other physiological molecules, such as the platelet-activating factor, in addition to alarmins, have been proposed as another preterm murine model (Wahid et al., 2020).

It is important to note that in most of these models, only the survival of neonates or inflammatory features of the fetus were analyzed, while other features already associated with preterm birth, like changes in the microbiota have not been assessed. This is surprising since, as commented previously, gut microbiota changes are present in preterm neonates. In this respect, Yu et al. (2016) used germ-free mice colonized with preterm human babies microbiota, and they found differences between mice colonized with low-weight preterm infants and mice colonized with normal-weight preterm infants in epithelium development, goblet cell numbers and Paneth cells. The main limitation of this study is that, even though specific pathogen free mouse microbiota is used as a control (which has better scores in general than preterm human microbiota), it lacks a term human microbiota control.

Although infection and inflammation are only a part of the events causing preterm delivery, most of the models developed to date are based on them. Another cause of preterm labor that is commonly addressed in murine models is the hormonal disbalance, including a few models of progesterone and prostaglandins (Romero et al., 2006; McCarthy et al., 2018; Arenas-Hernandez et al., 2019; Ginsberg et al., 2021). In the case of progesterone, it has been studied simultaneously with inflammation processes. In a model of *in vivo* T-cell activation through the intraperitoneal injection of α-CD3, progesterone improved the rate of preterm birth (Arenas-Hernandez et al., 2019). One of the main limitations of these models is that progesterone levels are different in mice and humans, mainly at the end of pregnancy, when humans present a decrement of this hormone, but rodents have a peak the last 2 days of pregnancy. Probably, that is the reason why hormonal preterm delivery is less common or more difficult to predict/evidence in humans (McCarthy et al., 2018). It has been described that progesterone might be related with the early softening of the uterine cervix, which can cause the luteolysis in rodents, but not in humans (Romero et al., 2006). In line with this, it is known that cervix structure and integrity is compromised in preterm delivery, but this has been addressed only in the context of inflammation in murine models. Partial cervical excision during a LPS-induced preterm labor murine model, caused even earlier preterm labor than only LPS treatment, mainly due to higher degradation of collagen in the cervix (Jeong et al., 2021).

Finally, some other non-inflammatory models of preterm birth have been developed, but compared with the models available for inflammation, they are scarce. These include C-section preterm delivery, premature rupture of membranes by the abnormal secretion of biglycan and decorin, uterine quiescence, endocannabinoid signaling, hyperhomocysteinemia, exposure to environmental stimulus, like tobacco or alcohol (McCarthy et al., 2018). Another environmental model addressed the importance of diet during pregnancy and found that the lack of vitamin D and calcium increased the rate of preterm births associated with changes in the placenta microstructure (Wilson et al., 2020).

All these models, that rarely analyzed the correct development of neonates, are mostly focused in the study of the causes of the preterm delivery than in studying the preterms. Furthermore, the outbreak of preterm delivery by an inflammatory insult does not allow to properly study the role of the inflammation in the natural preterm delivery.

## Preterm Delivery: Altered Primocolonisation, Hyperpermeability and Low-Grade Inflammation

An increased body of evidence supports that preterm birth results from a breakdown in fetal-maternal tolerance joint to an excessive premature inflammation (Green and Arck, 2020). This premature inflammation, together with other injurious conditions, contribute to the onset and perpetuation of a sustained inflammatory condition in the newborn (Humberg et al., 2020).

The origin of systemic inflammation is often the gut, as the most common metabolic compounds with potential to perpetuate the inflammation come from the gut microbiota (Humberg et al., 2020). The gut is a privileged place for the microbiota-host crosstalk interactions. In mammals, the microbial colonization of mucosal surfaces during the 1st days of life is fundamental to the development and education of the host immune system (Macpherson and Harris, 2004; Round and Mazmanian, 2009; Hooper et al., 2012). These interactions can have long-standing effects, such as encouraging environmental tolerance (including to food-based antigens and commensal bacteria) and/or contributing to the development of disease in later life (Marsland and Salami, 2015; Gensollen et al., 2016). It has been suggested that there is a critical period during early development in which disruptions in microbiota-host interactions could irreversibly harm the host priming, thus hampering the establishment of healthy homeostasis (Brugman et al., 2015). Such disruptions are a major cause of problems in the development of immunity and predispose the host to developing inflammatory diseases and altered gut barrier function (King et al., 2012; Shen and Wong, 2016). In particular, when microbiota-host crosstalk is perturbed during critical developmental periods in early life, chronic inflammation may result, leading to health issues such as inflammatory bowel diseases (IBD), allergies, and obesity (Garn et al., 2013; Li et al., 2014; Marsland and Salami, 2015).

The intestinal barrier is the first line of defense against external risks. It is composed by a functional unit conformed by a physical barrier of a mucus layer and a monolayer of epithelial cells and a functional layer composed mainly of immune cells. It separates the self from the non-self (Lopetuso et al., 2015). When the intestinal barrier is healthy, it allows selective paracellular transport, regulating solute and water fluxes while preventing the entry of bacteria and toxins. When barrier function is impaired, altered permeability and dysfunction can result, ultimately leading to health issues (Perrier and Corthésy, 2011; Camilleri, 2012). Furthermore, as hyperpermeability increases local antigen exposure, it could also activate the intestinal immune response and provoke inflammation (Natividad and Verdu, 2013). During early life, contrary to the adulthood, the passage of macromolecules is enhanced to allow immune system priming (Westrom et al., 2020). In normal conditions this hyperpermeability is controlled and decreases quickly during the 1st weeks of life due to the intestinal barrier maturation (Heinonen et al., 2018). This intestinal barrier maturation happens in correlation with a significant increase in microbiota diversity and it is positively promoted by early exclusive breastmilk feeding (Heinonen et al., 2018).

**FIGURE 1:**
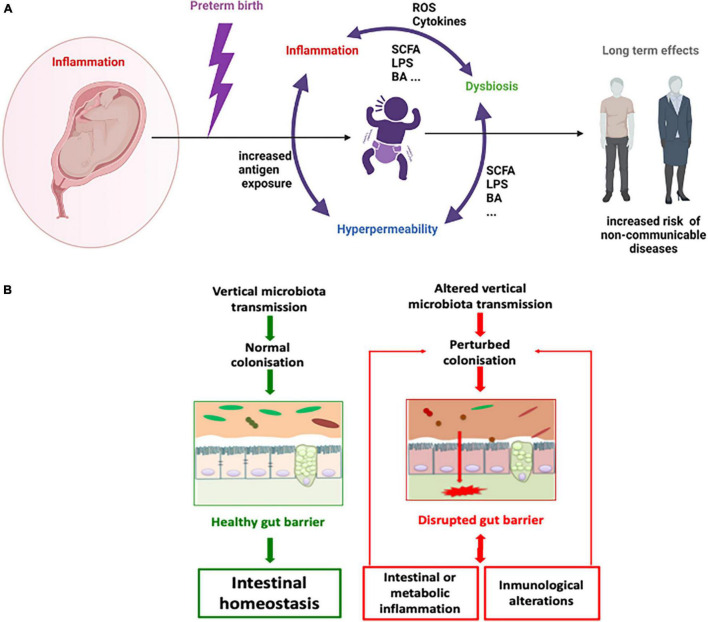
Microbial dysbiosis, inflammation and hyperpermeability vicious circle in the preterm context. A general schema **(A)** and a focus on gut barrier **(B)**. SCFA = short chain fatty acids, BA = bile acids, ROS = reactive oxygen species, LPS = liposaccharide.

As most interactions between the host and its microbiota take place at the gut barrier and primocolonisers are essential in the maturation of the epithelium (Tomas et al., 2013) our main hypothesis is that the disruption of primocolonisation causes a breakdown in homeostasis along the gut barrier that feeds a vicious circle in which intestinal and metabolic inflammation and immunological alterations result in inflammatory diseases. This mechanism could be the linchpin behind the phenotypic effects in preterms that lead to major disease susceptibility in later life (Figure 1).

**FIGURE 2:**
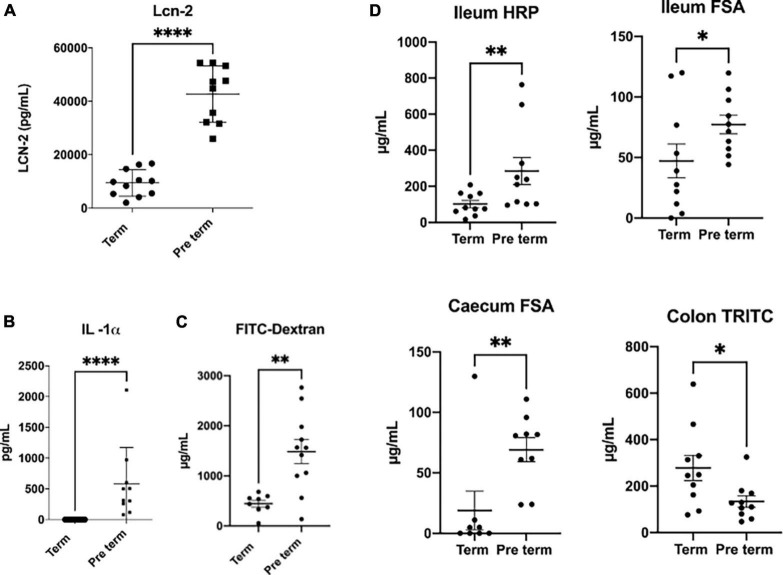
Comparison of inflammation and permeability parameters. Comparison between spontaneous Swiss-CD1 preterm pups (born at 20 days of gestation) and Swiss-CD1 term pups (21 days of gestation) at weaning (3 weeks of age). Lipocalin 2 (LCN-2) concentration in serum, measured by Elisa **(A)**. Systemic permeability measured by doing gavage with the fluorescent molecule FITC-dextran (average size of 3,5 KDa) 3 h and a half before the euthanasia of the mice and measuring the fluorescence in blood in a spectrophotometer **(B)**. Serum concentration of IL-1α measured by Biolegend plex **(C)**. Local permeability measured in Ussing chambers: ileum transcellular permeability measured by transfer of horseradish peroxidase (HRP), ileum paracellular permeability measured by sulfonic acid (FSA) passage and caecum and colon paracellular permeability measured by FSA or TRITC-dextran (TRITC) passage respectively **(D)**. *n* = 8-11, Man Whitney non-parametric test, *p < 0.05, ^**^p < 0.01, ^***^p < 0.001, ^*⁣*⁣**^p < 0.0001.

Taking advantage of a set of natural premature pups that were born in our animal facilities during another experiment setup, we decided to make a preliminary study and compare gut permeability features between these pups and another litter born at the usual time point for our mice strain (Swiss-CD1 mice). These mice were analysed at weaning (3 weeks of age for this murine strain). We measured a very sensitive general marker of systemic inflammation in serum (lipocalin 2 and the pro-inflammatory cytokine IL1-α) and the general permeability by using FITC-dextran marker (Figures 2A,B). Our results confirmed that preterm pups have a higher systemic inflammation and an increased general gastrointestinal permeability, although most of the cytokines tested were under the limit of detection. Besides, when permeability was measured at local level by mounting colon, ileum or caecum samples in Ussing Chambers we corroborate that the local permeability was altered (Figure 2D). These alterations are more complex than expected. Ileum samples of preterm pups showed an increased in paracellular and transcellular permeability (as measured by horseradish peroxidase and sulfonic acid transfer through the tissues respectively), while caecum samples only showed altered transcellular permeability. Surprisingly, a decrease in transcellular permeability for the preterm pups measured by the passage of TRITC-dextran was detected in colon samples (Figure 2C). This scenario confirmed that (i) altered intestinal permeability and systemic inflammation are present in natural preterm pups, even after 3 weeks of life, and (ii) the situation is probably most complex than expected and further analysis are required to decipher the situation. Unfortunately, the actual murine model options, based mostly in unchaining the preterm birth by inducing inflammation, do not allow to properly address the real role of inflammation and gut microbiota in this phenomenon.

## Discussion

Delivery is a huge revolution for the newborn. Even in normal conditions, the baby passes from the comfort of the intrauterine environment to the unfriendly external world. Human babies are considered as unmatured. There is a specific developmental sequence that allows the correct growth and development of the newborn. It means that, even in term babies, both physical and emotional development is required to progress toward maturity. This complex process is even more complicated in preterm babies (Haumont et al., 2012; Ansari et al., 2021). Although premature baby’s development happens in the same order as it would have happened in the womb, premature infants develop differently than full-term babies, as they are exposed to a different environment during this development and do not count with the same environmental support. In consequence, preterm babies are exposed to external microorganisms and insults with an immature immune and physiological status. Furthermore, preterm infant’s microbiota differs dramatically from that of term infants (Henderickx et al., 2019). These facts not only increase the risk of infection, but also dysregulate the microbiota-host crosstalk, which is fundamental for the achievement of a homeostatic condition (Brugman et al., 2015).

In the case of preterm birth, the immaturity of the immune and physiological systems seems to be a major driver of the alterations found, being inflammation and the consequent production of reactive oxygen species an elicitor of microbiota dysbiosis and gut hyperpermeability which in turn increase inflammation (Arboleya et al., 2012a). Nevertheless, the presence of an altered microbiota is also a major agitator by itself as it is able to induce oxidative stress, gut inflammation and hyperpermeability. To really understand the mechanism underlying the relation among all these factors, the development of new murine models that better mimics natural preterm birth and cover the study from a multidisciplinary point of view, including gut microbiota composition and functionality, gut barrier dysfunctions and immune system impairment, is required.

## Data Availability Statement

The raw data supporting conclusions of this article will be made available by the authors, without undue reservation.

## Ethics Statement

The animal study was reviewed and approved by local ethical committee for animal experiment (COMETHEA), INRAE Jouy en Josas, France.

## Author Contributions

SA, DR-C, MG, and RM designed the perspective and wrote the manuscript. RM and PL designed the experiments. RM and FM performed the experiments. SA, DR-C, PL, MG, and RM corrected the manuscript. All authors approved the last version of the manuscript and contributed to the article and approved the submitted version.

## Conflict of Interest

The authors declare that the research was conducted in the absence of any commercial or financial relationships that could be construed as a potential conflict of interest.

## Publisher’s Note

All claims expressed in this article are solely those of the authors and do not necessarily represent those of their affiliated organizations, or those of the publisher, the editors and the reviewers. Any product that may be evaluated in this article, or claim that may be made by its manufacturer, is not guaranteed or endorsed by the publisher.
